# Genes, inflammatory response, tolerance, and resistance to virus infections in migratory birds, bats, and rodents

**DOI:** 10.3389/fimmu.2023.1239572

**Published:** 2023-08-29

**Authors:** Patrick Douglas Corrêa Pereira, Daniel Guerreiro Diniz, Emanuel Ramos da Costa, Nara Gyzely de Morais Magalhães, Anderson de Jesus Falcão da Silva, Jéssica Gizele Sousa Leite, Natan Ibraim Pires Almeida, Kelle de Nazaré Cunha, Mauro André Damasceno de Melo, Pedro Fernando da Costa Vasconcelos, José Antonio Picanço Diniz, Dora Brites, Daniel Clive Anthony, Cristovam Wanderley Picanço Diniz, Cristovam Guerreiro-Diniz

**Affiliations:** ^1^ Ciência e Tecnologia do Pará, Laboratório de Biologia Molecular e Neuroecologia, Instituto Federal de Educação, Bragança, Pará, Brazil; ^2^ Seção de Hepatologia, Laboratório de Microscopia Eletrônica, Instituto Evandro Chagas, Belém, Pará, Brazil; ^3^ Instituto de Ciências Biológicas, Hospital Universitário João de Barros Barreto, Laboratório de Investigações em Neurodegeneração e Infecção, Universidade Federal do Pará, Belém, Pará, Brazil; ^4^ Centro de Ciências Biológicas e da Saúde, Universidade do Estado do Pará, Belém, Pará, Brazil; ^5^ Seção de Arbovirologia e Febres Hemorrágicas, Instituto Evandro Chagas, Ananindeua, Pará, Brazil; ^6^ Research Institute for Medicines (iMed.ULisboa), Faculty of Pharmacy, Universidade de Lisboa, Lisbon, Portugal; ^7^ Department of Pharmaceutical Sciences and Medicines, Faculty of Pharmacy, Universidade de Lisboa, Lisbon, Portugal; ^8^ Department of Pharmacology, Laboratory of Experimental Neuropathology, University of Oxford, Oxford, United Kingdom

**Keywords:** virome, disease tolerance, resistance, inflammatory response, sickness behavior

## Abstract

Normally, the host immunological response to viral infection is coordinated to restore homeostasis and protect the individual from possible tissue damage. The two major approaches are adopted by the host to deal with the pathogen: resistance or tolerance. The nature of the responses often differs between species and between individuals of the same species. Resistance includes innate and adaptive immune responses to control virus replication. Disease tolerance relies on the immune response allowing the coexistence of infections in the host with minimal or no clinical signs, while maintaining sufficient viral replication for transmission. Here, we compared the virome of bats, rodents and migratory birds and the molecular mechanisms underlying symptomatic and asymptomatic disease progression. We also explore the influence of the host physiology and environmental influences on RNA virus expression and how it impacts on the whole brain transcriptome of seemingly healthy semipalmated sandpiper (*Calidris pusilla*) and spotted sandpiper (*Actitis macularius*). Three time points throughout the year were selected to understand the importance of longitudinal surveys in the characterization of the virome. We finally revisited evidence that upstream and downstream regulation of the inflammatory response is, respectively, associated with resistance and tolerance to viral infections.

## 1 Introduction

The devastating Severe Acute Respiratory Syndrome Coronavirus 2 (SARS-CoV-2) pandemic has killed more than 6.88 million people worldwide [for details see COVID 19 online data https://www.worldometers.info/coronavirus/and (1, 2)]. SARS-CoV-2 was first identified in China, in December 2019 (World Health Organization, WHO, March 28, 2023). On March 11, 2020, WHO declared COVID-19 a pandemic, and on December 18, 2020, Pfizer/BioNTech vaccines and Moderna received emergency use authorization from the Food and Drug Administration (FDA). However, by this time, the virus had already caused 1.7 million deaths and had spread to 216 countries (3). Globally, there have been severe knock-on effects on the economy, owing to the restrictive measures put in place to attempt to prevent SARS-CoV-2 from spreading, which have increased unemployment, poverty, hunger, and inequalities (4), particularly in low and middle income countries (5–10).

Arguably, the extent the pandemic was controlled by the administration of non-replicating RNA-vaccines that were designed to induce immunoprotection in survivors (11–14).

If the ongoing pandemic could have been anticipated, few scientists would have expected the magnitude and speed of its spread (15) or the number of people that are still experiencing post-COVID-19 syndrome or post-acute sequelae of SARS-Cov-2 (16) and still need ongoing medical attention (17–19). These individuals regularly require the input of costly multidisciplinary teams for individualized rehabilitation and therapy (20, 21).

The Pandemic has encouraged government throughout the world to prepare public health policies to anticipate zoonotic diseases and those transmitted by arthropods, the main causes of worldwide outbreaks (22). Ongoing international efforts indicate that the most likely profile for the next pandemic will be similar to the last where another zoonotic RNA virus passes from non-human animal to humans and then to sustained human-to-human transmission (23, 24).

Understanding interspecies viral transmission and the ability to model the environmental factors that contribute to the spread of viral diseases to humans is central to the prevention of future pandemics (25). To begin to understand these processes, a broader characterization of the animal virome and an improved understanding of the host response to the presence of the virus is essential (26, 27). Unbiased metagenomic next-generation sequencing (mNGS) is helping to shed new light on virus evolution (28). mNGS applied to bats (29), migratory birds (30, 31), rodents (32) and arthropods (33) to characterize their viromes, and the transcriptomic analysis of gene expression of these large groups of non-human animals is well underway. Here we reanalyzed the virome of bats, rodents and birds to search for mechanistic insight into tolerance and resistance to virus infection. We also highlight the importance of understanding and regulating a dysfunctional innate immune system and inflammasome to control tissue damage (34).

## 2 Virus reservoirs in mammals and birds

In the wild, an enormous number of viral species circulate in specific niches and very little is known about their biology and transmission (35–39). Many of these viral species have a severe impact on livelihoods and the trade in livestock (40). The commercial trade in wildlife that is associated with hunting and consumption of wild animals has been recognized as an important route for the emergence of human zoonotic diseases (41, 42).

In a viral ‘reservoir’ species there is normally an organized immunological response to the virus, but often there is no apparent disease activity (43–46). In these situations, there is usually a low level of viral infection, and the host is able to tolerate some viral replication. Indeed, as mentioned above, many viral ‘passengers’ are carried by hosts for a lifetime (47).

### 2.1 The immune system of the bat: a prototypic model for virus tolerance

The pandemic placed bat immunology in the spotlight and it has been suggested that further detailed study may help prevent future pandemics (48). Bats harbor many viruses and share human and domestic animal environments. They have a long-life expectancy and their diversity and winged locomotion favors the emergence and dispersal of new viral species (49). As bats are the only autonomous flying mammals, they are, possibly, the only non-human mammals to occupy almost all regions of the planet, except the Arctic and Antarctic poles (50).

Bats may (like any other mammal) become sick when infected by certain viral species, and the spread of new pathogens into bat colonies be fatal for them (51). However, previous studies on the diversity and evolution of viruses in bats have revealed that they exhibit unique immunological approaches to enable coexistence with viral infections, showing minimal or no overt clinical signs, while allowing enough replication for transmission (26, 52–57). Many of these bat-borne viruses induce several zoonotic human diseases (e.g. Ebola virus, Marburg virus, Nipah virus (NiV), Hendra virus (HeV), SARS-coronavirus (SARS-CoV), MERS coronavirus and (MERS-CoV) (38, 58–60) and all viral species associated with these diseases were found in apparently healthy bats (61).

Experimental infection with Egyptian rousette bats with Marburg virus (MARV) or Ebola virus followed by mRNA expression analysis, revealed the concentration of virus transcripts in the liver and many transcriptional changes in multiple tissues (62). These tissue changes included a robust overexpression of the ANXA1 gene, involved in the regulation of inflammation and cell signaling pathways (63), as well as of MRC1 (CD206) gene associated to a subset of tissue resident macrophages regulating inflammation (64). MARV severe disease includes inflammatory gene dysregulation (cytokine storm) and immune response suppression followed by systemic damage and death in humans, which is avoided in the Egyptian rousette bats by the upregulation of antiviral genes IFN-stimulated gene 15 (ISG15), IFN Induced Protein With Tetratricopeptide Repeats 1 (IFIT1) and 2′,5′-oligoadenylate synthase (OAS) 3 gene in the absence of any significant induction of proinflammatory genes, such as C-C Motif Chemokine Ligand 8 (CCL8), FAS (also called CD95 or APO-1 or TNFRSF6) and interleukin(IL)-6 (27). Because these viruses do not elicit a robust immune responses, it seems to prevent immunopathology, but it also prevents viral clearance that (65) leads to immunological tolerance. Thus, while it has been suggested that viral zoonotic risk seems to be homogeneous among mammalian and avian reservoirs hosts (66), it has been demonstrated that bats harbor significantly higher proportion of zoonotic viruses than all other mammalian orders (49, 67). A broad metagenomic analysis, recently conducted in China, to screen the virome in pharyngeal and anal swab samples of 4440 apparently healthy bats (no overt signs of disease) from 40 major different bat species revealed the presence of a diverse set of viruses (59). The most widely distributed families of mammalian viruses found were *Herpesviridae, Papillomaviridae, Retroviridae, Adenoviridae* and *Astroviridae* (~61% of the total viral sequence reads). Many reads related to other families were also found and included *Circoviridae, Paramyxoviridae, Coronaviridae, Caliciviridae, Polyomaviridae, Rhabdoviridae, Hepeviridae, Bunyaviridae, Reoviridae, Flaviviridae and Picornaviridae*, and the subfamily *Parvovirinae*. These authors identified in the swabs of a few genera, *Rhinolophus* spp. *Miniopterus* spp., and *Myotis* spp., 16, 10 and 13 virus families respectively, suggesting that these three genera are the major reservoirs for mammalian viruses in China (59).

These data confirmed previous findings that bats are indeed unique reservoirs of diverse virus with immune tolerance (15, 61, 68).

The ability of bats to control intracellular pathogens does not mean that they are not susceptible to extracellular infections (69). Indeed, environmental exposure of small, hibernating brown bats to *Pseudogymnoascus destructans* has been shown to cause infection and high mortality in colonies associated with white nose syndrome (70).

### 2.2 Migratory birds, tolerance, resistance, and virus long-distance dissemination

Free-living migratory birds can also be zoonotic reservoirs and can contribute to the dispersion of microorganisms as biological carriers, mechanical carriers, or carriers of infected hematophagous ectoparasites (71–73). As for bats, migratory birds also host viruses and can be the long-distance transmitters or local amplifiers of a variety of viruses that may induce severe disease in humans, domestic animals, and other wildlife (74–78).

Virome investigations of free-living birds and exploration of the impact of environmental factors on viral infection in the hosts may help to detect virus species with potential translation into human risk (79, 80). In keeping with these ideas, the virome of cloacal swab specimens collected from 3182 birds including more than 87 different species (mostly wild species), from 10 different avian orders was investigated (81). They identified 707 viral genomes from 18 defined families and 4 unclassified virus groups, with 265 virus genomes comprising new virus families, genera, or species. The authors provided evidence for the potential cross-species transmission of certain viruses in wild birds, showing > 95% amino acid sequence identity with previously reported viruses in domestic birds.

Several avian species sharing the variants of the Influenza A virus, except H17N10 and H18N11 subtypes, have been isolated exclusively from bats (82). Influenza viruses of types A and B lead to seasonal influenza epidemics, but only type A is linked to pandemics (83, 84). Migratory birds directly contribute to the maintenance and dissemination of avian influenza A virus within the Northern Atlantic flyway of North America (85).

Owing to the detection of rearrangements and mutations of the Influenza virus in wild birds, public concern about the potential pandemic risk posed by this viral species and its mutations is growing significantly (77, 86, 87). Indeed, evidence of transmission from avian viral species to humans has previously been described for A/H7N9, A/H6N1 and A/H10N8 variants, and approximately 35% of patients infected with A/H7N9 succumbed to zoonotic infection (87).

Although most people with influenza exhibit acute respiratory symptoms and muscle aches, with or without fever, which disappear within 1 week (88), avian species do not always have symptomatic infection, facilitating human exposure to the virus. Wild birds also play significant roles in the ecology and circulation of Eastern and Western Equine Encephalomyelitis and Sindbis alphaviruses, West Nile, Usutu, and St. Louis Encephalitis flaviviruses (71, 89, 90).

The inflammatory response to viral infections in migratory birds has also been well documented in several different domestic and aquatic birds (91). Gallinaceous poultry species, domestic ducks and various aquatic and terrestrial birds are vulnerable to avian influenza viruses (92–96) and host inflammatory response seems to contribute to morbidity and mortality in all species investigated so far (96, 97). Avian influenza (AI) viruses for example, have been detected in more than 105 species of birds of different taxa but the species most frequently found harboring influenza virus belong to Charadriiforms (gulls, terns and shorebirds) and Anseriformes (ducks, geese and swans) orders, where no apparent disease signs or lesions were found (97). It has been suggested that the recognition and elimination of invading pathogens (resistance)(98, 99) or the control of the infection associated tissue damage (tolerance)(100) may explain asymptomatic or minimally symptomatic infections.

Virus infections were associated with liver diseases in chickens raised in scaled farms causing significant economic losses and this was associated with multiple virus infections. Indeed, panvirome profiling of livers, spleens, kidneys, and recta revealed coinfection of chicken infectious anemia virus, avian leukemia, avian encephalomyelitis virus (AEV) and multiple fowl adenoviruses, with a higher abundance of the last two virus species in the liver (101). Many other virus infections with novel strains and variants caused economic losses to the poultry industry worldwide and these included infectious bronchitis virus (102), bursal disease virus (103), H5Nx avian influenza viruses in Europe, Africa and the Americas (104–106), H5N8 and H5N1 avian influenza viruses (107–111). Similarly, the host response to pigeon paramyxovirus type I (PPMV-1) infection (associated with New Castle disease, a great threat to the pigeon industry), induces strong innate immune responses and intense inflammatory responses at an early stage and this is associated with viral pathogenesis (112). After infection, pigeons showed upregulated expression of toll-like receptors (TLRs), such as TLR2, TLR3 and TLR15, together with interferon (IFN) gamma (IFNɣ) and IL-6, whereas IL-18 expression was found down- regulated (112). PPMV-1 is now worldwide disseminated causing extensive infections in domestic and feral pigeons, wild birds and poultry (113).

Differential host responses to avian influenza viruses in avian species with differing susceptibilities were analyzed by transcriptomics, and susceptible birds showed strong neuro-inflammatory responses associated with greater viral load (114). Ducks are natural hosts and reservoirs of influenza A virus and transcriptomic analysis in infected individuals showed this condition associated with downregulation of a distinct set of proinflammatory cytokines in lung, key elements of leukocyte recruitment and complement in intestine (115).

Comparative analysis of chickens and ducks following high pathogenic avian influenza virus infection was associated with increased proinflammatory responses in chickens whereas downregulation of inflammatory response in ducks was associated with mild or asymptomatic profiles (116). In line with these observations mallard ducks are permissive to low pathogenic avian influenza viruses in their intestinal tissues without overt disease signs, thus limiting the duration of proinflammatory cytokine expression, whereas in chickens, respiratory and systemic disease is associated with enhanced virus replication and associated tissue damage (117). While the co-evolution of Mallard ducks with influenza virus have allowed host-pathogen interaction and resistance through retinoic acid-inducible gene I (RIG-I) pathway, providing asymptomatic or minimally symptomatic infections with robust IFN response, chickens lacking the key sensors of RIG-I pathway, with compromised host response, show significant inflammatory changes, associated clinical signs and lesions to the skin, respiratory, digestive, reproductive and nervous systems following high pathogenic influenza virus (HPAIV) infections (117–119).

### 2.3 Tolerance and resistance to virus infections in rodents

Rodentia is the most diverse order of mammals, representing more than 40% of mammalian species including 33 families and 2,600 species (120). They are natural virus reservoirs of a variety of species associated with human severe diseases (121–124).

At the molecular level, Cohn and collaborators performed transcriptional and physiological monitoring across 33 mouse strains during *in vivo* infection with the influenza virus (125). They identified two host-defense gene programs associated with disease tolerance and resistance respectively, providing a paradigm for exploring these immune responses in different species.

At the cellular level, a key subset of specialized T cells for regulation of immune responses and maintenance of the immune tolerance in the periphery, the Treg cells, are recruited and expanded, undergoing functional and molecular reprogramming (126). These changes may protect against exacerbated immune responses while providing tissue homeostasis (127).

Dual RNAseq differential expression analysis following HIN1 infection in two mouse strains with contrasting resistance to influenza virus infections enabled investigation of both the viral and host gene expression profile in the same individual and revealed that host genes were involved in the development of severe pathology, viral replication and immune responses (128). From this report it emerges that IFN *Regulatory Factor 7 (Irf7) gene* deletion (obtained in the knock-out line on a C57BL/6J background) was associated with greater susceptibility to H1N1 infection with significant body weight losses and increased mortality compared to wild controls (128).

Furthermore, it has been demonstrated using lymphocytic choriomeningitis and Influenza A virus models in mice, that the Treg-dependent immunosuppression is associated with the effector state of Treg cells, which maintain stability and functionality while exposed to IFNɣ. The stability of regulatory T cells may be diminished or reinforced by different Type-1 cytokines (127). The deletion restricted to regulatory T (Treg) cells of the IFNɣ receptor, but not to the IL-12 receptor, prevents Th1-type polarization and promotes Th2-type polarization, avoiding long inflammatory process, and adequate response from memory T cells (129).

It is important to highlight, however, that although many significant immunological discoveries have been obtained in laboratory mice, they do not directly mimic the physiology of wild mice living in their natural environment. To circumscribe this limitation, a new murine model named “wilding” was generated by transferring embryos of C57BL/6 into wild mice (130). This approach revealed that the immune profiles of wilding mice, laboratory mice, and wild-type mice showed that wilding individuals and laboratory mice exhibit a different epithelial barrier microbiome, gut microbiome, virome, and repertoire of pathogens. Interestingly, immunological phenotypes of wildings and wild-type mice were closer to and seemingly a better mimic of the human immune system. In agreement, the first phase 1 clinical trial in multiple laboratory mouse models of autoimmune/inflammatory diseases, using CD28 monoclonal antibodies, which are able to activate regulatory T cells, resulted in human life threatening activation of inflammatory T cells and cytokine storms (131). Similarly, laboratory mice born from wild mice (wilding mice) after treatment with a CD28-superagonist showed an inflammatory cytokine response and an absence of Treg expansion (130).

### 2.4 Lessons from arthropods

In addition to the sustained virome surveillance of bats, rodents and migratory birds, arthropods are another essential source of information for the future control of pandemics (132–134). As in mammals and birds, arthropods also react to potential threatens with an innate immune system response comprising both cellular and humoral components (135). Arthropods harbor numerous arboviruses that may spread through migratory birds and bats, at stopover sites, to other species, or to other individuals of the same species, and many of these arboviruses are of great epidemiological interest (136, 137). For example, mosquito-borne and tick-borne viruses cause some of the most severe diseases with high fatality rates in humans and animals and continue to threat human and livestock health globally (135, 138, 139).

To understand the emergence of arboviruses and the dynamics of potential outbreaks, it is essential to reconstruct the virome of the species of interest and understand its composition and potential modulatory actions on arbovirus transmission (140, 141). For example, mosquito vector competence for dengue is modulated by insect-specific viruses (142). Using virome analysis of 815 urban Aedes mosquitoes collected from 12 countries worldwide it was demonstrated that two mosquito-specific viruses were the most abundant (Phasi Charoen-like virus and Humaita Tubiacanga virus). The spatiotemporal analysis of virus circulation in an endemic urban area revealed a 200% increase in chances of having DENV in wild *A. aegypti* mosquitoes when both Phasi Charoen-like virus and Humaita Tubiacanga virus were present (142).

Metagenomic analysis of mosquitoes can provide valuable information, including new or unsuspected arboviruses, as well as non-arboviral pathogens ingested from hosts on which they feed (143). Next generation sequencing of RNA extracted from excreta of *Aedes vigilax* and *Culex annulirostris* reversed for cDNA and sequenced, allowed the assembly of near-full-length viromes of Australian Anopheles totivirus, Wuhan insect virus 33, and Hubei odonate virus 5 and 7 new viruses, closely related to members of the order *Picornavirales* and to previously described, but unclassified RNA viruses (143).

At least 500 arboviruses have been identified so far, 100 of which may induce human diseases and 40 of them cause domestic animal diseases (144, 145), and the Amazon is a spotlight for emergence of new arboviruses and this has been associated with disturbance of the natural ecosystem (146).

Emergent viruses may involve urbanization - in which humans have become the amplification hosts - and peridomestic mosquitoes (mainly Aedes aegypti) mediate human-to-human transmission (dengue, yellow fever, chikungunya and Zika viruses) (147, 148). Secondary amplification, for example, has occurred in outbreaks of Japanese encephalitis, Venezuelan equine encephalitis, and Rift Valley fever viruses (149, 150). Alternatively, simple spillover from enzootic cycles may occur as it is observed in West Nile fever (151), Crimean-Congo hemorrhagic fever (152, 153), tick-borne encephalitis (154, 155) and Alkhurma hemorrhagic fever virus (156, 157).

Vector-borne viruses enter the vector, infect, and replicate. Escaping from the gut and reaching the salivary glands, they infect vertebrate host through saliva during blood feeding (136, 158, 159). Sialotranscriptomes of unfed and fed ticks showed that blood feeding alters the expression profiles to facilitate the feeding process and pathogen transmission (160).

## 3 The influence of host physiology and environmental influences on virome

Human pathogens often originate from other species that act as reservoirs/hosts and from where eventual spillover and sustained human-to-human transmission takes place (161). Indeed, around 89% of the 180 recognized RNA viruses with the potential to harm humans are zoonotic (162) and the global effort for emergent infectious disease surveillance and investigation is currently in the wrong place and must migrate to hotspots in lower latitudes, such as tropical Africa, Latin America and Asia (163). The spread of humans into new ecosystems owing to the requirement for more land, climate change, loss of biodiversity, and the trade and consumption of wild animals have all enhanced the risk of transmission of these pathogens from animals to humans and increases the likelihood of pandemics (41, 164, 165).

To identify the intermediate host from where viruses may spillover, pathogen screening of animals, animal products and their movements as well as diagnostic of emerging infectious disease is required along the human-wildlife interface (157, 162). In these surveillance studies (birds, bats, rodents or arthropods viromes), the collection of ecological and physiological parameters in different environments and at different times throughout the year is also essential to understand whether a host response is resistant or tolerant of a virus (166). Indeed, host and environmental factors throughout the year influence seasonal virus transmission and highlighting the importance of longitudinal surveys at multiple time points in many hosts (167). Stopovers in bird may contribute to the recovery of constitutive immune function, which is compromised during migration (168). For example a non-stop transatlantic flight may not allow the innate immune system to recover before arrival at the final destination and may affect the ability of the bird, in this instance, to clear a virus (169).

Virome descriptions are generally based on cross-sectional space-time sampling limited to a single time point (166, 167), thus precluding how intraspecific variation and host physiology and ecology affect viral communities (170). As few reports have highlighted the importance of these influences on virome studies, as an exemplar, we show here how viral material in the brain of two long-distance migratory birds with contrasting migratory flights (*Calidris pusilla* and *Actitis macularius*) has provided an exciting opportunity to explore phenotypic variations influencing host physiology and environmental factors on the virome. The semipalmated sandpiper (*C. pusilla*) leaves the Bay of Fundy in Canada and arrives on the coast of South America 5-6 days later in a single non-stop flight with no opportunity to feed (171). While the *Actitis macularius* arrives at the wintering places with overland flights, after multiple stopovers for feeding and rest (migratory map at https://explorer.audubon.org/explore/species/1566/spotted-sandpiper/migration?sidebar=collapse).

We compared differential gene expression of viral species quantifying virus transcripts in the brain of different individuals of these trans-oceanic and trans-continental overland long-distance migratory birds. All individuals were captured within three-time windows of the wintering period. Four birds were collected between September and October (recently arrived birds), four between April and May (pre-migratory birds) and four in the intermediate time window (wintering birds). All birds were collected in Otelina Island (0°45’42.57”S and 46°55’51.86”W), in the mangrove area of the Amazon estuary at Bragança municipality, Brazil.

We searched for virus transcripts using the pipeline VIRTUS (v.1.2.1) and VIRTUS2 (v.2.0) using as reference the genome and transcriptome of *Calidris pugnax* (Accession code ASM143184v1) followed by NCBI Ref-Seq Viral Genomes (172).

**Figure 1:**
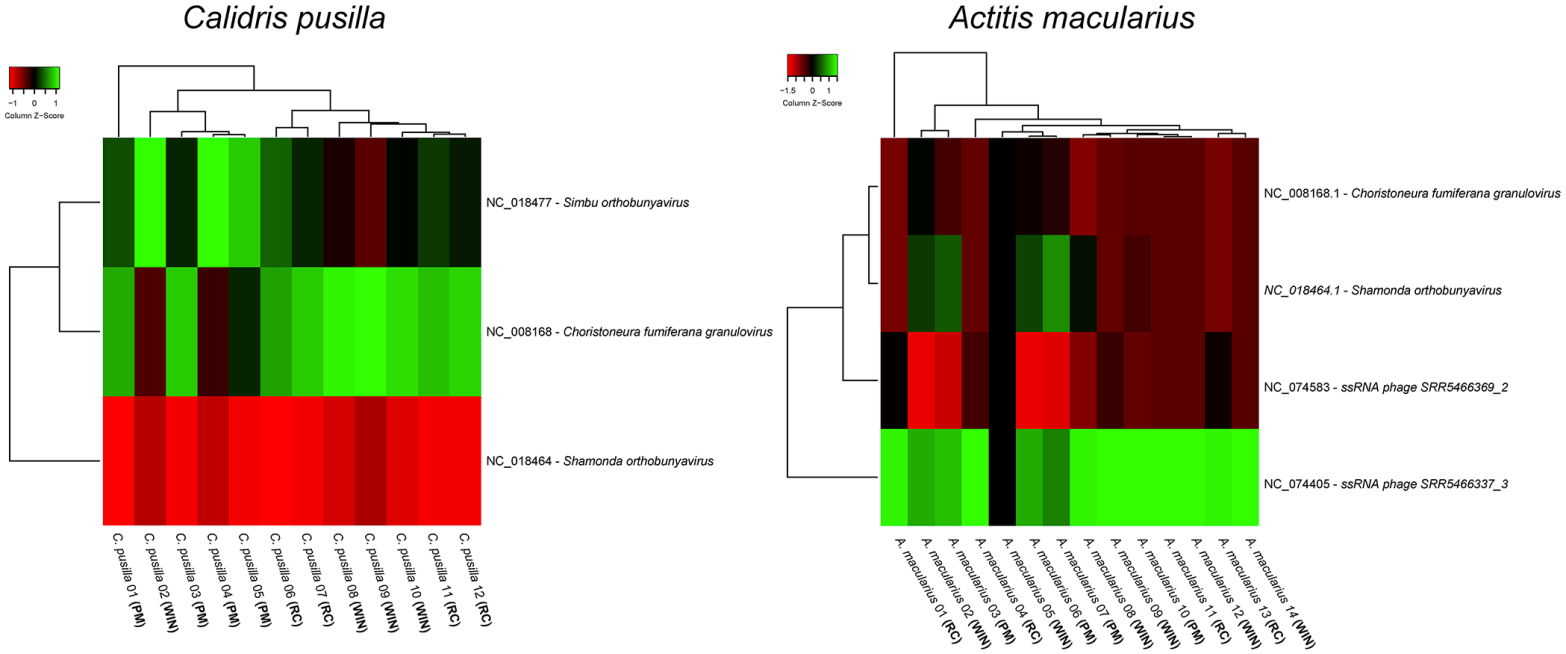
Differential expression of virus transcripts in the whole brain transcriptome of different birds captured at three-time windows of the wintering period: recently arrived, intermediate (wintering) and premigratory. Note the existence of marked differences in the heat maps of *Actitis macularius* (left) and *Calidris pusilla* (right). We divided the wintering period into three equal time windows: newcomers (August–November), wintering (December–March), and pre-migration (April–July). RC = recently arrived birds (captured between August and November); PM = premigratory birds (captured between April and July); WIN = wintering birds (captured between December and March).

*C. pusilla* and *A. macularius* telencephalon transcriptomes (FASTQ files) subjected to VIRTUS 2 (v.2.0) pipeline, revealed the presence of 370 and 626 virus species respectively (see **Supplementary Tables S1, S2**). The differential virus transcripts expressions were quantified with VIRTUS (v.1.2.1) by comparison of gene expressions in the three time points: recently arrived, intermediate and pre-migratory birds are shown in **Figure 1**. **Table S3** indicates capture dates for each bird.

While stopover may contribute to recover constitutive immune function, that is compromised during migration (168), non-stop transatlantic flight may not allow innate immune recovering before arrival (169).

In addition, adaptive mutations in the viral genome over time as it occurred with SARS-CoV-2, may result in the emergence of multiple variants which may alter virus interactions with host cell receptors for better or worse (173, 174).

## 4 Avoidance, resistance, and tolerance to viruses

Avoiding, resisting, or tolerating viruses are different mechanisms that ultimately must act to protect organisms from the homeostatic associated imbalance owing to infections (98, 99).

Avoidance requires that the pathogen must be detected before host infection and this may be acquired through social information and chemical signals to recognize infected conspecifics (175–177). Olfactory cues that induce aversive behavior exude from animals with acute inflammatory processes or infected, pushing away their conspecifics (178, 179). Formyl peptide receptor 3 is a G protein-coupled receptor expressed in subsets of sensory neurons of murine vomeronasal organ that act as pathogen sensor (180). Indeed, Fpr3 expression in the immune system is upregulated after lipopolysaccharide and bacterial MgrB peptide, driving avoidance behavior through the stimulation of a subset of vomeronasal sensory neurons (179).

The primary function of the immune system is, however, the recognition and elimination of invading pathogens (resistance) (98, 99) or alternatively, the control of the damage induced by a given burden (tolerance) (100).

When the pathogen is a virus species, its early detection in host cells is accompanied by activation of antiviral effector mechanisms, including type I IFN production (181, 182), natural killer (NK) cell activation (183), and B and T cell responses (184, 185).

Bats, as other eutherian mammals, synthesize anti-viral effector molecules including type I IFN, T and B cell responses, and innate responses to pathogen-associated molecular patterns (PAMPs) derived from viruses, bacteria or parasites (61, 186, 187). RNA sequencing in tissues and cells from different bats confirmed, at molecular level, these similarities (188), and this included receptors and molecules associated with innate and adaptive immunity, and microRNAs (189).

The IFN system provides the first line of defense against viral infection in vertebrates with type I IFN promoting humoral immunity (190) and both type I and III IFNs associated with the adoption of an anti-viral state in infected and neighboring cells (53, 191). However, the innate and adaptive immune systems that detect and respond to invading pathogens in eutherian mammals must be integrated to guarantee efficient protection. The role of integrating these two systems in both bats and humans belongs to the lymphocytes of the innate immune response, which share both adaptive and innate features, and are abundant in the peripheral circulation and in barrier tissues (192, 193).

Innate lymphocytes (“unconventional” or “innate-like T cells”), including innate lymphoid cells, natural killer cells, ɣδ T cells and mucosal-associated invariant T cells (194), were detected in a number of bat species (192).

Sequencing, assembly and the analysis of the genome of *Rousettus aegyptiacus* (Marburg virus reservoir) demonstrated the differential expansion of NK cell receptors, MHC class I genes, and type I IFN, as compared to other mammals, suggesting an inhibitory immune state (tolerance), rather than enhanced antiviral defenses (resistance) in bat immune system (195). However, many aspects of innate and adaptive immune responses of bats differ from human response, and these include a reduction of several immunoglobulin subclasses and contraction (fewer IFN genes compared with any other sequenced mammal genomes) of the type I IFN locus, and unusual constitutive expression of IFN-α in tissues and cells, unaffected by viral infection. This unique constitutive expression of IFN-stimulated subset of genes associated with anti-viral activity and resistance to DNA damage, may contribute to the coexistence of bats with viruses (53).

As previously described, humans and bats react in contrasting ways to the presence of SARS-CoV-2 (15, 196). While the inflammatory response is the major cause of deaths associated with the SARS-CoV-2 infection in humans, infected bats downregulate the genes associated with the inflammatory response avoiding its deleterious effects (26, 61, 197, 198).

Macrophages of the bat *Myotis myotis* and the domestic mouse (*Mus musculus*) when stimulated with polycytidylic acid, Poly (I:C) (ligand of toll-like receptor 3 – TLR3) mimicking a virus infection, or lipopolysaccharides from bacterial membrane (ligands of toll-like receptor 4 - TLR4) showed high levels of mRNA of IFNβ, tumor necrosis factor (TNF) and IL-1β. However, the bats exhibited sustained high-level transcription of the anti-inflammatory cytokine IL-10, which was not detected in the house mouse (197).

In addition, bats produce the innate potent IFN-inducible restriction factor tetherin that restricts the replication of diverse enveloped viruses (including retro-, filo-, herpes- and arenaviruses). As a consequence, the release of budding virions from infected cells may be inhibited (199–201) that would normally stimulate antiviral IFN response through the nuclear-factor kappa B (NF-KB) signaling pathway (202, 203).

### 4.1 Disease progression and dysfunctional inflammatory response to virus infection

More than two years after its emergence, the SARS-CoV-2 virus continues to affect a subgroup of patients. These patients, who have recovered from the acute phase of the disease, report a diverse repertoire of symptoms that characterize chronic or long COVID-19 (204, 205). During the acute phase, as part of the virus replicative cycle, SARS-CoV-2 induces the death and injury of virus-infected cells and tissues, followed by a wave of local inflammation associated with increased secretion of the pro-inflammatory cytokines and chemokines IL-6, IFNɣ, MCP1 (monocyte chemoattractant protein-1) and IP-10 (interferon gamma inducible protein) (206). In most individuals, the recruited cells clear the infection in the lung, the immune response subsides, and patients recover (207). However, in some patients, a dysfunctional immune response occurs, that is characterized by augmented cytokine production, that leads to widespread lung inflammation and multi-organ failure (207–209).

Indeed, severe acute respiratory syndrome induced by SARS-CoV-2 induces proinflammatory immune responses in the periphery and/or in the brain *via* classical TL receptor inflammatory pathway and this event is crucial for the onset of acute and chronic neuroinflammation and sickness-related behavior (210). Dysregulated immune responses resulting in lymphopenia and increased proinflammatory cytokine production seems to be the principal cause of the pathophysiology in COVID-19 and patients. High concentrations of IL-6 accompany the most severe cases (211).

The humoral immune response by B-lymphocytes is part of the adaptive immune mechanisms, controlling viral infections through the neutralizing antibodies and antibody-dependent cellular toxicity (212, 213). The elimination of viral infection and the apoptosis of virally infected cells is also provided by B lymphocytes through effector molecules, exhibiting antibody-independent functions (214). However, lack of B cells has been associated with a mild form of COVID-19 suggesting that inflammatory cytokines, especially IL-6, may have a central role in the disease severity (213). In fact, patients with agammaglobulinemia (caused by a lack of B lymphocytes), show mild symptoms, short duration, and no need of treatment by IL-6 inhibitors, while patients exhibiting variable immune deficiencies with dysfunctional B lymphocytes, (also showing significant low levels of immunoglobulins in the serum), show severe outcomes and require mechanical ventilation, antiretroviral agents and IL-6-blocking agents (215).

Survivors from acute phase may exhibit a form of post-acute sequelae characterized by atypical chronic persistence of symptoms that may last for months resulted in the long-COVID syndrome (204). Long COVID appears to be a multi-organ disease and with a spectrum of symptoms (systemic, neuropsychiatric, cardiac, and respiratory) have been associated with persistent inflammation, induced autoimmunity, and putative organ reservoirs of SARS-CoV-2 RNA or antigens (216–219). Recent evidence supports, however, the hypothesis that the persistence of the inflammatory response for a long period after the acute phase is closely correlated with the post-acute sequelae of COVID-19 (PASC). Actually, the combination of digital epidemiology with selective biobanking recent analysis suggested that instead of autoantibodies, the elevated plasma levels of IL-6, IL-1β and TNF-α is in the core of the clinical symptoms of PASC COVID-19 (220). While rheumatoid factor, antinuclear antibodies, and antiphospholipid antibodies showed no correlation with post-acute COVID-19 sequelae, analysis of blood samples from participants with ongoing post-COVID19 sequelae, revealed at 6 and 8 months after infection, increased levels of the pro-inflammatory cytokines TNF-α, IL-1β and IL-6 in close correlation with symptoms of the post-acute COVID-19 syndrome (220).

### 4.2 Virus infections, NLRP3-mediated inflammation, RIG-I, ISGs, SOCs and JAK STAT

#### 4.2.1 NLR family pyrin domain-containing protein 3

The important inflammasome sensor, NLR family pyrin domain containing protein 3 (NLRP3), has been linked to both viral-induced and age-related inflammation (221). Inflammasomes activate inflammatory caspases promoting the maturation of IL-1β and IL-18, while inducing cell death by pyroptosis (222). Dysregulation of the inflammasome is associated with several autoinflammatory syndromes and autoimmune diseases. Interestingly, ASC2 that negatively regulates inflammasome activation in both sterile and infectious settings was identified in myeloid cell genomes of 13 bat species, whereas none or minimal expression was found in most human tissues (223). Indeed, bats dampen inflammation by enhanced innate immune tolerance with implications for longevity and unique viral reservoir status (221). In contrast, the NLRP3 inflammasome was found activated in response to SARS-CoV-2 infection and identified in peripheral blood mononuclear cells (PBMCs) and postmortem tissues of COVID-19 patients (224). Moreover, NLRP3 inflammasome activation and hyperinflammation is associated with COVID-19 severity, and Pan and collaborators demonstrated that SARS-CoV-2 nucleocapsid protein can directly interact with NLRP3 to promote the assembly and activation of NLRP3 inflammasome (225). Though some inflammasome genes were also expressed in birds, little is known about the role of inflammasomes in avian responses (226). However, we may speculate that NLRP3 inflammasome is a potential target for therapy. Some similarities between the inflammasomes of humans, rodents, and other animal species suggest that the development of veterinary therapeutics may consider the inflammasome modulation by small molecules once the recombinant proteins used for human therapeutics are not economically practical. Recently, it has been demonstrated that ACS2 also suppresses SARS-CoV-2-immune-complex-induced inflammasome activation (101). In recent years, food bioactive compounds, in particular fruit and vegetables rich in flavonoids and antioxidants, as NLRP3 inflammasome modulators, have been discussed as a whole, rather than a single nutrient or functional compound (227). However, there is a lack of human data on their activity on the NLRP3 activation and there is a widespread opinion that the inhibition of the inflammasome activation may only have a minimal effect, and would only contribute more significantly if an autoimmune response is already established (228).

#### 4.2.2 Retinoic acid-inducible gene I

Virus species must introduce their RNA or DNA genomes into the infected cell to replicate, and a number of nuclei acid sensors (229) including the retinoic acid-inducible gene I (RIG-I)-like receptors (RLRs) are activated leading to convergent signaling cascade and transcriptional expression of genes encoding Type I IFNs (230, 231). RLRs are RNA sensor molecules that play essential roles in innate antiviral immunity (232) Type I IFNs bind to IFN-α/β receptor (IFNAR) which activates the Janus kinases (JAKs) and the signal transducer and activator of transcription (STAT) 1 and 2, which in turn lead to the expression of ISGs with a variety of anti-virus effects (231).

Frequent spillover of animal influenza viruses to humans, including, swine influenza viruses (233) and avian influenza virus (234) represents risks of zoonotic outbreak and pandemic (235, 236).

Birds are important reservoirs for RNA viruses and the innate defense against RNA viruses in birds involves detection of viral RNA including RIG-1 receptors, endosomal TLRs and their downstream adaptor proteins (237). Aquatic birds and shorebirds are common synanthropic species most commonly associated with avian influenza viruses (238). Because of the complete absence of any detectable RIG-I sequences in several galliform species, including the domestic fowl (Gallus gallus) (239), waterfowl and shorebirds that occasionally are associated with poultry facilities may become a source of significant damage to poultry farming (238).

Although RIG-I is involved mainly in antiviral signaling activated by RNA viruses, DNA virus infections (e.g. duck virus enteritis virus) may also increase the expression of IFN- β and RIG-I (240). RIG-I in infected cells by influenza A binds to virus genome and these single-stranded viral genomes are the natural RIG-I agonists that triggers a cascade of events leading to antiviral cytokines production (241). In contrast, the Middle East respiratory syndrome coronavirus (MERS-CoV), strongly inhibits both MDA5- and RIG-I-mediated activation of IFN-β promoter activity, while downstream signaling molecules are left largely unaffected (242, 243).

RIG-I and MDA5 (melanoma differentiation-associated gene 5), collectively known as RLRs, differentially recognized viral double-stranded RNA virus. For example, RIG-I seems to be essential for IFNs production in response to paramyxoviruses (244), influenza A virus (245) and Japanese encephalitis virus (246, 247), whereas MDA5 is critical for picornavirus detection (248) which is confirmed by high susceptibility of RIG-I-/- and MDA5-/- mice to these viral infections (249).

Because of the bat immune system has the ability to clear or maintain a number of viral infections without apparent clinical signs, bat cell lines have been used to characterize the IFN response to different viral infections in bat reservoirs (52, 250–253). Following infection with Marburg virus (MARV) and Ebola virus (EBOV) Egyptian fruit bats survived with no overt disease (254, 255) and it was demonstrated that MARV and EBOV replication was inhibited in the Egyptian fruit bat cell line R06EJ, transfected with Egyptian fruit bats innate immune genes. The overexpression of Type I, II and III IFNs, as well as DDX58 (RIG-I), IFH1 and IRF1 were associated with virus inhibition and maybe responsible for viral clearance (256).

#### 4.2.3 IFN stimulated genes

IFNs act as extracellular cytokines activating cell surface receptors followed by intracellular kinase signal transduction and phosphorylation of transcription factors leading to the induction of ISGs allowing antiviral defense, antiproliferative activities, and stimulation of adaptive immunity (257). All type I IFNs, type II IFN (IFNɣ) and type III IFNs exhibit well-described viral inhibitory properties (258–261).

Infectious bronchitis virus (IBV) is a single strand, positive sense RNA virus (belonging to gammacoronaviruses) (262) that induces infectious bronchitis and is responsible for significant economic losses within the global poultry industry (263). Infectious bronchitis is a good example of the effects of IFN-I suppression where IBV – encoded nucleocapsid protein acts as an antagonist of IFN-I, compromising the expression of IFN-stimulated genes allowing IBV evasion from avian innate immune responses (264).

The host innate immune response mediated by IFN-1 results in the up-regulation of hundreds of ISGs and provides early protection against viral infection. Comparative studies have revealed that while many ISGs are common to all mammals, each species displays its own distinct repertoire of ISGs (265). Antiviral IFNs are responsible for the high tolerance of bats to zoonotic viruses (266), with reduced inflammatory phenotypes and bat species-specific adaptations affecting innate immune responses, where genomic and functional studies revealed unique subsets of ISGs (53, 265). For example, camelids and bats are tolerant to MERS-CoV replication and display no signs of sickness behavior. When the cervical lymph node cells from MERS-CoV convalescent llamas were pulsed with viral strains (clades B and C), although viral replication was not supported in the lymph node cells, a cellular immune response was mounted. Th1 responses (IFN-γ, IL-2, IL-12) with a transient peak of antiviral responses (type I IFNs, IFN-λ3, ISGs, PRRs and TFs). In addition, significant expression of inflammatory cytokines (TNF-α, IL-1β, IL-6, IL-8) and inflammasome components (NLRP3, CASP1, PYCARD) were suppressed (267). It was suggested that IFN-λ3 counterbalances the inflammatory processes and integrate innate and adaptive immune responses.

Recent studies in bats indicate that the unique balance between host defense and immune tolerance may explain why bats are so special virus reservoirs (15, 195, 221, 268). Previous studies demonstrate that the innate immune system of unstimulated bat tissues remain switched on due to constitutive expression of three IFN-α gene that limit viral replication without the presence of high antibodies titre (53, 65, 268). Moreover it has been demonstrated that bat microbiome from Great Himalayan Leaf-nosed bats (*Hipposideros armiger*) transplanted into H1N1 infected mice, reduces the inflammatory response and increases survival rate being that associated with increased production of flavonoid and isoflavones as well as with the quick innate immune response induced by the bat fecal transplanted microbiota, thus conferring mouse tolerance to influenza virus (H1N1) infection (269).

#### 4.2.4 SOCS and JAK STAT pathways

Cytokines are secreted glycoproteins that act as intercellular messengers essential for proliferation, differentiation and growth or apoptosis of the target cells, being these process initiated after binding to cell surface receptors and activation of intracellular cascades including the JAK-STAT pathway (270, 271). Most cytokines use JAK and signal transducers and activators of transcription (JAK/STAT) pathway to promote gene transcriptional regulation resulting in the expression of hematopoietic growth factors and immunomodulatory and inflammatory cytokines (271). Their signals are attenuated by multiple mechanisms and these include the suppressor cytokine signaling (SOCS) family of proteins, which is the principal negative regulation mechanism for the JAK-STAT pathway (272). An important negative-feedback inhibitor of signaling induced by cytokines that act *via* the JAK/STAT pathway is the SOCS family of proteins (273). When a virus infects the host cells, the innate immune receptors identify, distinguish, and react to the invader using nucleic acid or viral protein sensors (274) inducing infected cells to produce type I IFNs and proinflammatory cytokines (275). Cytokines stimulate the expression of SOCS proteins, intracellular inducible inhibitors that limit the signal magnitude of cytokines employing JAK and STAT pathways (276, 277) or target ubiquitinated signal transduction factors, avoiding potential tissue damage caused by excessive secretion of cytokines, maintaining homeostasis (275). Several viruses, however, have developed mechanisms to induce robust host SOCS protein expression increasing inhibition of protective antiviral signaling pathways allowing viruses to evade host immune response (276, 278). Indeed, the hijacking and subsequent upregulation of the SOCS proteins upon viral infection, suppress the associated JAK-STAT signaling activities, followed by reduction of the host antiviral response and viral replication (279). SOCS proteins increased expression was found in many viruses including SARS-CoV (279), herpes simplex virus (280), hepatitis B virus (281), hepatitis C virus (282), Zika virus (283), respiratory syncytial virus (284) and influenza A virus (285).

Overexpression of SOCS1 leads to a decrease in phosphorylation levels of JAK1, tyrosine kinase 2 (TYK2) while also inhibits antiviral and antiproliferative responses induced by IFN-I. Consistent with this, SOCS1 ablated cells and SOCS1−/− mice are resistant to viral infection (286; 275). Similarly, porcine reproductive and respiratory syndrome virus (PRRSV) (major threaten to global swine industry) increases SOCS3 production *via* activation of p38/AP-1 signaling pathway to promote viral replication and persistent infection (287).To obtain a favorable outcome for viral infections, avoiding the installation of an inflammatory pathology with eventual fatal evolution, the host must have strict control of its expression (288, 289). For this, the innate immune system recognizes molecules that identify many pathogens associated molecular patterns (PAMPs) or damaged associated molecular patterns (DAMPs) and initiates a rapid response producing inflammatory mediators that activate programmed cell death pathways including pyroptosis, apoptosis and necroptosis with significant crosstalk among them (290–292).

## 5 Concluding remarks

Few scientists would have expected the magnitude and speed of the spread of the COVID-19 pandemic. While the deaths, rightly, have attracted the headlines, an unknown number of carriers of post-acute sequelae of SARS-CoV-2 still require medical attention. To anticipate pandemics in the future and avoid unnecessary morbidity and mortality, it has been suggested that a broader characterization of the animal virome, host physiology and the underlying mechanisms associated with disease tolerance and anti-viral defenses are essential. We reviewed the literature concerning the virome of bats, rodents and migratory birds and highlighted the potential mechanisms of tolerance and resistance to virus infection in these groups. It is now clear that the type of host inflammatory response to the presence of virus is central to these mechanisms. We also emphasize the importance of understanding and controlling the dysfunctional innate immune system and inflammasome to deal with future outbreaks in the period before we have effective vaccines.

## Author contributions

CD, CG-D, PP, DD, NM and MM contributed to conception and design of the study. PP organized the database and performed statistical analysis. DD optimized figures and adapted submission to the journal requirements. CD wrote the first draft. DB wrote section of the manuscript. CD, DA, DD and DB contributed to final edition. All authors contributed to the article and approved the submitted version.
