# Supplementary material for: Genes, inflammatory response, tolerance, and resistance to virus infections in migratory birds, bats, and rodents
Source: Front Immunol. 2023 Aug 29;14:1239572. doi: 10.3389/fimmu.2023.1239572 (PMC10497949; doi:10.3389/fimmu.2023.1239572)
Supplement: Supplementary file 3 [file Table_3.docx]

Supplementary Table 3: Capture dates of *Actitis macularius* and *Calidris pusilla* individuals used for virus discoveries.

| Specie | Experimental Group | Sample ID | Capture date |
| --- | --- | --- | --- |
| *Actitis macularius* | Recently arrived | *A. macularius* 14 | Aug-31-2019 |
|  |  | *A. macularius* 05 | Aug-31-2019 |
|  |  | *A. macularius* 02 | Aug-31-2019 |
|  |  | *A. macularius* 08 | Aug-31-2019 |
|  |  | *A. macularius* 09 | Aug-31-2019 |
|  |  | *A. macularius* 12 | Aug-31-2019 |
|  | Wintering | *A. macularius* 13 | Jan-06-2019 |
|  |  | *A. macularius* 11 | Jan-06-2019 |
|  |  | *A. macularius* 04 | Jan-06-2019 |
|  |  | *A. macularius* 01 | Jan-06-2019 |
|  | Pre-migratory | *A. macularius* 06 | May-10-2021 |
|  |  | *A. macularius* 07 | May-10-2021 |
|  |  | *A. macularius* 10 | May-10-2021 |
|  |  | *A. macularius* 03 | May-10-2021 |
| *Calidris pusilla* | Recently arrived | *C. pusilla* 11 | Sep-12-2015 |
|  |  | *C. pusilla* 06 | Sep-13-2015 |
|  |  | *C. pusilla* 07 | Oct-13-2015 |
|  |  | *C. pusilla* 12 | Oct-13-2015 |
|  | Wintering | *C. pusilla* 09 | Feb-07-2015 |
|  |  | *C. pusilla* 08 | Feb-07-2015 |
|  |  | *C. pusilla* 02 | Feb-07-2015 |
|  |  | *C. pusilla* 10 | Feb-07-2015 |
|  | Pre-migratory | *C. pusilla* 04 | Apr-15-2018 |
|  |  | *C. pusilla* 05 | May-17-2018 |
|  |  | *C. pusilla* 01 | May-17-2018 |
|  |  | *C. pusilla* 03 | May-17-2018 |
